# Improving Outcomes Through Personalized Recommendations in a Remote Diabetes Monitoring Program: Observational Study

**DOI:** 10.2196/33329

**Published:** 2022-03-21

**Authors:** Sowmya Kamath, Karthik Kappaganthu, Stefanie Painter, Anmol Madan

**Affiliations:** 1 Teladoc Health Purchase, NY United States

**Keywords:** personalization, type 2 diabetes, recommendation, causal, observational, mobile health, machine learning, engagement, glycemic control, mHealth, recommender systems

## Abstract

**Background:**

Diabetes management is complex, and program personalization has been identified to enhance engagement and clinical outcomes in diabetes management programs. However, 50% of individuals living with diabetes are unable to achieve glycemic control, presenting a gap in the delivery of self-management education and behavior change. Machine learning and recommender systems, which have been used within the health care setting, could be a feasible application for diabetes management programs to provide a personalized user experience and improve user engagement and outcomes.

**Objective:**

This study aims to evaluate machine learning models using member-level engagements to predict improvement in estimated A_1c_ and develop personalized action recommendations within a remote diabetes monitoring program to improve clinical outcomes.

**Methods:**

A retrospective study of Livongo for Diabetes member engagement data was analyzed within five action categories (interacting with a coach, reading education content, self-monitoring blood glucose level, tracking physical activity, and monitoring nutrition) to build a member-level model to predict if a specific type and level of engagement could lead to improved estimated A_1c_ for members with type 2 diabetes. Engagement and improvement in estimated A_1c_ can be correlated; therefore, the doubly robust learning method was used to model the heterogeneous treatment effect of action engagement on improvements in estimated A_1c_.

**Results:**

The treatment effect was successfully computed within the five action categories on estimated A_1c_ reduction for each member. Results show interaction with coaches and self-monitoring blood glucose levels were the actions that resulted in the highest average decrease in estimated A_1c_ (1.7% and 1.4%, respectively) and were the most recommended actions for 54% of the population. However, these were found to not be the optimal interventions for all members; 46% of members were predicted to have better outcomes with one of the other three interventions. Members who engaged with their recommended actions had on average a 0.8% larger reduction in estimated A_1c_ than those who did not engage in recommended actions within the first 3 months of the program.

**Conclusions:**

Personalized action recommendations using heterogeneous treatment effects to compute the impact of member actions can reduce estimated A_1c_ and be a valuable tool for diabetes management programs in encouraging members toward actions to improve clinical outcomes.

## Introduction

Diabetes is a chronic progressive disease affecting 34 million Americans with 1.5 million newly diagnosed each year [1,2]. Individuals living with diabetes are at greater risk of health complications including increased hospitalizations that result in 1 of every 4 health care dollars spent on diabetes-related care in the United States [3]. An essential factor in successfully living with diabetes is effective self-management, which has shown to improve glycemic control and reduce hospital admissions and the overall lifetime cost of health care [4].

Diabetes self-management efficacy and improved glycemic control is supported by programs that offer education, coaching, glucose monitoring, and physical activity [5-7]. Diabetes management programs have shown to be as or more effective than usual care in providing a significant reduction in hemoglobin A_1c_ (HbA_1c_) [8-10]. Additionally, structured self-monitoring of blood glucose (SMBG) has been observed to improve glycemic variability and provide greater self-efficacy in management by helping an individual understand lifestyle behaviors’ impact on blood glucose (BG) values over time [2,11]. However, while advances in diabetes treatment options, diabetes management programs, new technologies to support self-management, and the rise in digital health are rushing the market, half of individuals with diabetes have an HbA_1c_ value of 7.0% or higher and struggle to obtain consistent glycemic control [1]. This alludes to potential gaps in self-management programs, technology, and delivery to the individual [12].

Personalization has been identified as a key tool in digital health to enhance user engagement for improved outcomes, which is often a missing factor in the development of diabetes digital health programs [12]. Self-management best practices and user preference must be taken into consideration to effectively provide a personalized experience within a diabetes management program. This study has proposed and analyzed the feasibility of using heterogeneous treatment effect models for personalizing action recommendations within a digital remote diabetes monitoring program (RDMP).

## Methods

### Livongo for Diabetes

Livongo for Diabetes is an RDMP focused on empowering members with education and tools to self-manage their diabetes through mobile technology. The program offers members a cellular-enabled, two-way messaging device that measures BG and delivers personalized insights into their glycemic management; free unlimited BG test strips; real-time support from diabetes response specialists 24 hours a day, 7 days a week, 365 days a year; and access to certified diabetes care and education specialists (CDCESs) for support and goal setting.

Livongo members’ glucose meter use was captured remotely through the cellular-enabled device. Members also had access to a mobile phone app that tracked historical SMBG readings and provided reminders for SMBG checking, physical activity, and food log tracking; asynchronous chat with coaches; ability to schedule private coaching sessions with CDCES; educational content for diabetes self-management; and allowed members to send historical reports of SMBG readings to care providers, family members, and friends.

### Study Design

A retrospective feasibility study was conducted to compute heterogeneous treatment effect for five different action categories in the reduction of estimated A_1c_ for members enrolled in Livongo for Diabetes with type 2 diabetes and to identify which actions could be most effective for each member. Within each action category, members were classified into a treatment or control group defined by engagement level. The effectiveness of each action category was assessed by computing the heterogeneous treatment effect for each action category for each member.

### Population Selection

**Figure 1:**
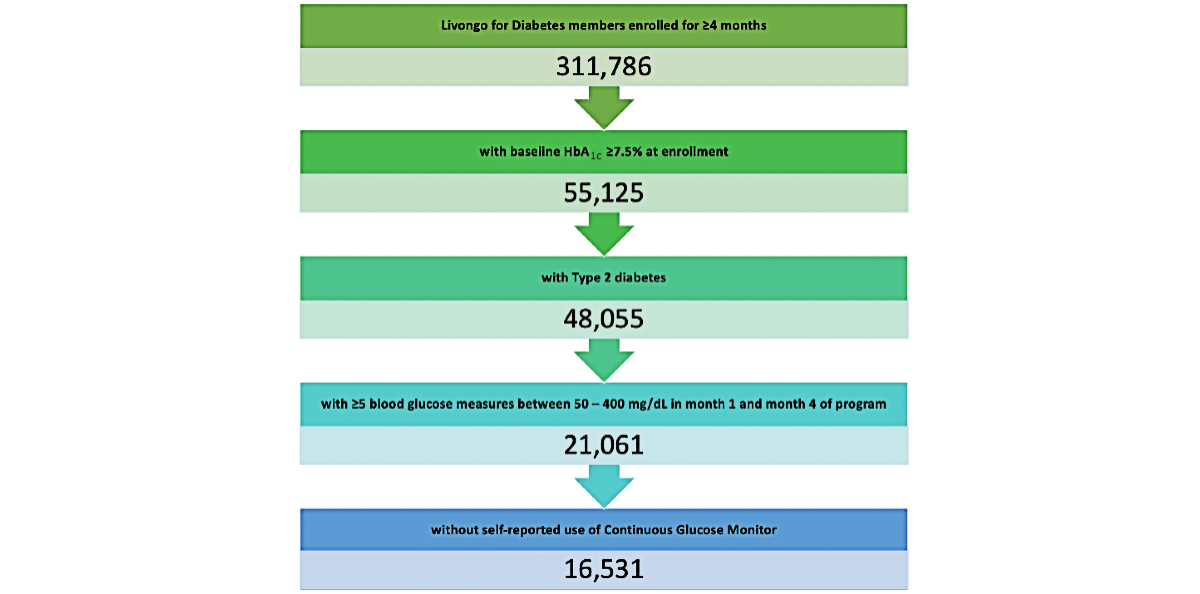
Study population funnel with inclusion and exclusion criteria. HbA_1c_: hemoglobin A_1c_.

Members enrolled in Livongo for Diabetes for a minimum of 4 months with a baseline estimated A_1c_ ≥7.5% at 30 days post enrollment were included in the study. Additional inclusion criteria were a self-reported diagnosis of type 2 diabetes at enrollment, ≥5 SMBG measures between 50 and 400 mg/dL in month one and month four of their program, and had not self-reported the use of a continuous glucose monitor (see Figure 1). Member demographics, self-reported preferences around communication and health-related interests, and level of engagement with various program features within 3 months following enrollment were used as covariates for modeling outcomes.

### Ethics Approval

The institutional review board approval was granted by Aspire IRB (#520160099), and guidelines outlined in the Declaration of Helsinki were followed.

### Action Categories

Various program features were categorized and grouped to form five action categories, otherwise known as interventions per the causal inference formulations (see Textbox 1 for action category descriptions).

**Action category name and descriptions.**

**Monitoring**

Number of self-monitoring blood glucose checks a member performed on the device

**Coaching**

Number of scheduled certified diabetes care and education specialist coaching sessions or asynchronous chat with a coach

**Physical activity**

Physical activity recorded using synced steps data

**Nutrition**

Members engagement with nutrition- and meal plan–related nudge recommendations and food log

**Content**

Members’ engagement with educational content nudge recommendations

### Measures

#### Estimated A_1c_

Estimated A_1c_ was calculated using the A_1c_-derived average glucose model where estimated A_1c_ = [mean BG over past 30 days + 46.7] / 28.7 [13]. Mean BG was calculated using SMBG values gathered through the member’s device.

The intervention outcome, *Y*, is defined as the difference between estimated A_1c_ in month four and month one post enrollment for members with a starting self-reported HbA_1c_ ≥7.5%. Therefore, members with a more negative *Y* have a better clinical outcome.

### Model Features

Treatment effect was modelled using self-reported member information and member engagement during the first 3 months post enrollment. The following variables were used as covariates in the model:

- Demographics: age, gender, BMI, race
- Self-reported medical information: self-reported HbA_1c_ at enrollment, diabetes management level of self-efficacy, insulin use, on oral diabetes meds, received flu vaccine, smoking behavior
- Self-reported preferences: preferred channels of communication, interest level in becoming active and healthy
- Engagement: average days between Livongo website use; average days between Livongo mobile app use; number of days of Livongo mobile app use; average days between SMBG checks; estimated A_1c_ at month two and three; days with SMBG hypoglycemia readings in month one, two, and three; days with SMBG hyperglycemia readings in month one, two, and three

### Computing Heterogeneous Treatment Effect

The sample was defined to be composed of members with covariates, *X*, in treatment or control cohorts, denoted by *T=t_1_* or *t_0_*, respectively, and intervention outcome, *Y*. Ideally, treatment effect, *τ*, would be measured for each member as:

τ (t_0_, t_1_, x) = E[Y (t_1_) – Y (t_0_) | X=x ]

However, this was not possible in a real-world data set where a member could only be in one cohort, treatment or control, at a time and not in both. Therefore, observed samples were assumed to be from a joint distribution modeled by the equations:

Y = g (T, X) and T = f (X)

and the treatment effect was expressed as:

τ (t_0_, t_1_, x) = E[g(t_1_, X) – g(t_0_, X) | X=x ]

where *g* (*T*, *X*) denoted the likelihood of outcome for a member given an intervention, and *f* (*X*) denoted propensity of a member to be in the treatment or control cohort of the intervention.

With the assumption that all potential confounders were observed, the heterogeneous treatment effect for each member was computed using the doubly robust (DR) learning algorithm [14-17]. Treatment effect was computed by the DR algorithm using three different models. The first model performed regression on *[T, X]* to predict outcome *Y*. The second model preformed classification on *X* to predict *T*. Lastly, the two models are combined to compute the heterogenous treatment effect where the estimated outcome from the regression model is debiased by adding the inverse propensity weighted model residual. This method provided robust predictions with only one of the two predictive models needed to have a small error to obtain an unbiased treatment effect estimator [18].

### Engagement Thresholds for Control and Treatment Cohort Assignment

Engagement level was used to split data into control and treatment groups. The application of the DR learning algorithm enabled intervention outcomes from the treatment and control groups to be representative of the same population because the propensity model, *f* (*X*), incorporated any differences in population while computing the treatment effect.

Member engagement was measured for each action category during the initial 3 months post enrollment. The level of member engagement with each action category was used to assign members into the treatment or control groups through a defined threshold. If a member had higher engagement than the threshold, then the member was assigned to the treatment cohort, and the members who did not achieve the threshold were assigned to the control cohort. The treatment and control split only included member’s engagement in the action category of the program. Members in the control cohort received communication from Livongo in the form of emails and newsletters.

The engagement thresholds were defined independently for each action category. The threshold value impacted the size imbalance between the control and treatment groups, thereby affecting noise in the data set and consequently the model performance. For this reason, engagement thresholds for each action category were selected that minimize the modelling error while optimizing treatment effect.

## Results

### Modeling Heterogeneous Treatment Effect for Coaching Intervention

Treatment effect for the five actions categories were modelled independently. The action category of coaching is used to detail the process of selecting an engagement threshold and evaluate the heterogenous treatment effect model. Treatment effects across all action categories are then reported, followed by a proposed method to personalize action recommendations to optimize clinical outcomes.

**Figure 2:**
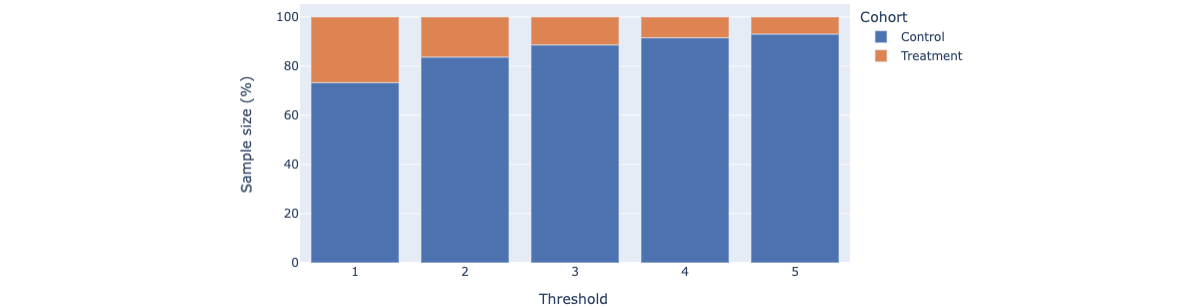
Control and treatment cohort sample size for different minimum number of coaching sessions defined as engagement threshold.

Members who completed sufficient scheduled coaching sessions or asynchronous coaching chat sessions were assigned to the treatment cohort, with members not meeting the criteria assigned to control. This intervention engagement threshold was observed to have an impact on the DR model performance. As the threshold increases, so does the control-treatment size imbalance and noise in the data. The control-treatment size imbalance for different thresholds is shown in Figure 2.

For a member to be considered as receiving treatment, the data is split into training and validation data sets, which was split by a ratio of 65:35. The treatment effect was modelled with a forest DR learner algorithm using a gradient boosting classifier and a random forest regressor to model the likelihood of the outcome, *g*, and treatment propensity, *f*, respectively. The mean squared error (MSE) of the model was a good indicator of confidence in predicting treatment effect. A threshold value that optimizes treatment effect while having lower MSE and sufficient sample size was selected for treatment and control assignment.

**Figure 3:**
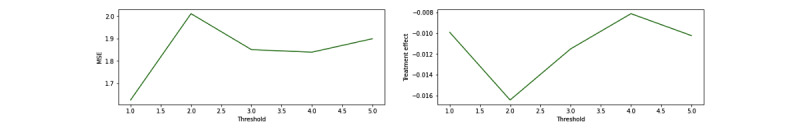
Top left: MSE of the doubly robust model to predict treatment effect of coaching intervention for different number of coaching sessions threshold. Top right: predicted treatment effect of coaching intervention for different thresholds. MSE: mean squared error.

The MSE of the heterogenous treatment effect estimator model for different thresholds and computed average treatment effect are shown in Figure 3. Based on Figures 2 and 3, a threshold of at least 3 coaching sessions (scheduled or chat sessions) within the initial 3 months post enrollment was used to assign members to the treatment cohort.

**Figure 4:**
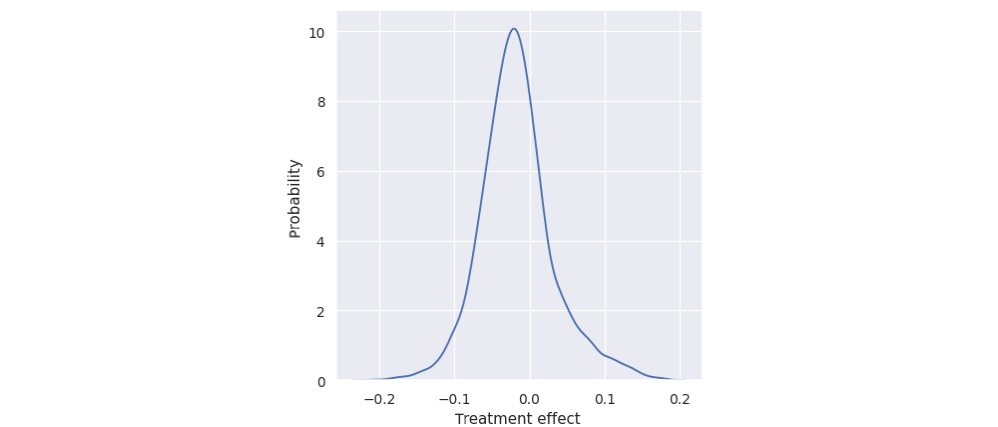
Distribution of computed treatment effects of coaching intervention.

Treatment effect of the coaching action category computed with the DR learner algorithm shows most members having a negative treatment effect, therefore, promoting a greater impact of coaching on estimated A_1c_ (see Figure 4). Members with positive treatment effects were those who the intervention did not improve the outcome.

### Evaluation of Heterogenous Treatment Effect Model for Coaching Intervention

A direct evaluation of causal models cannot be made on observational data where the true treatment effect is not known due to an inability to observe the effect of being treated or not for a particular sample simultaneously. Our causal model performance was evaluated indirectly by comparing the cumulative gain of the outcome when members are ranked by model prediction when compared to random sorting [19].

Cumulative gain is cumulative uplift multiplied by sample size, where uplift is defined as the difference between average outcomes of treatment and control cohorts. A model that performs well will have large uplift values in the first quantiles and decreasing values for larger ones. By comparing the cumulative gain of members sorted by treatment effects and randomly sorted, model performance can be inferred. The higher the area under the uplift curve (AUUC) in prediction when compared to random assignment, the better the model prediction.

**Figure 5:**
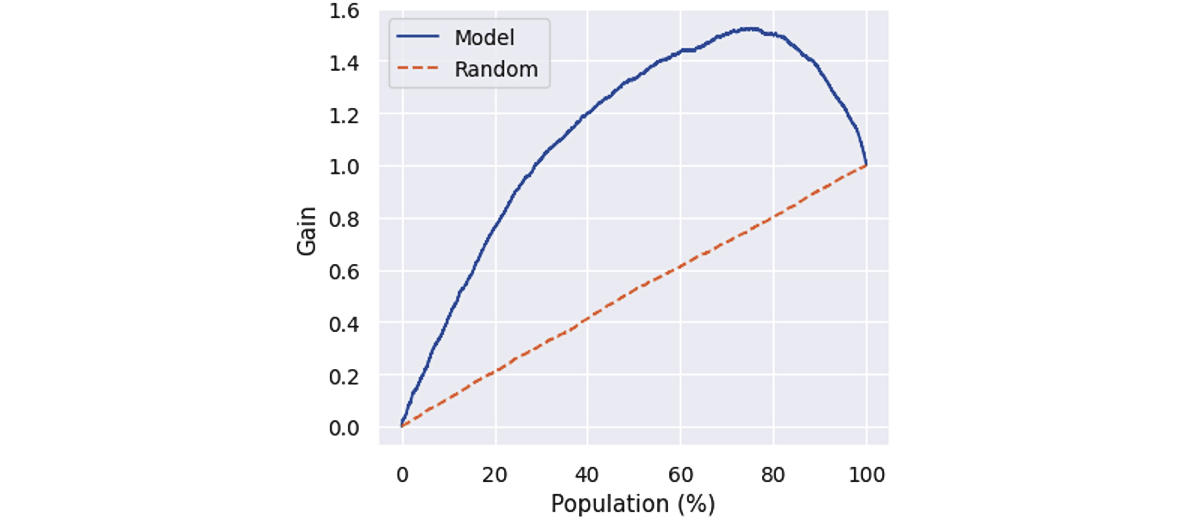
Cumulative gain of coaching intervention when members are sorted by predicted treatment effect (solid blue line) and with random sorting (dashed orange line). The cumulative gain plotted is the negative of uplift from intervention outcome variable (change in A_1c_) so that higher gain values reflect better results.

The cumulative gain in the outcome for the coaching action category when members are ranked by model predicted treatment effect and when randomly ordered is shown in Figure 5. The cumulative gain curves plotted are the negative of the outcome variables; therefore, the higher gain values reflect better results. The AUUC score for random assignment and assignment using the inferred treatment effect are 0.5 and 1.1, respectively.

### Modeling Heterogeneous Treatment Effect for Five Action Categories

The control-treatment cohort assignment for each of the five action categories for each member was inferred independently. From these independent analyses, engagement thresholds were defined for the five action categories optimizing treatment effect and sample size while lowering model MSE (see Textbox 2). The treatment and control cohorts vary across interventions depending on if they satisfied the engagement threshold condition for that intervention.

**Engagement thresholds within 90 days of enrollment for treatment.**

**Monitoring**

≥70 days with self-monitoring blood glucose checks

**Coaching**

≥3 coaching sessions (scheduled coaching sessions or asynchronous chat sessions with coach)

**Physical activity**

≥30 days with 2000 daily steps

**Nutrition**

≥2 food logs or ≥50% yes responses to nutrition-related nudges

**Content**

≥50% yes responses to content-related nudges

### Action Category Distribution and Outcomes

**Figure 6:**
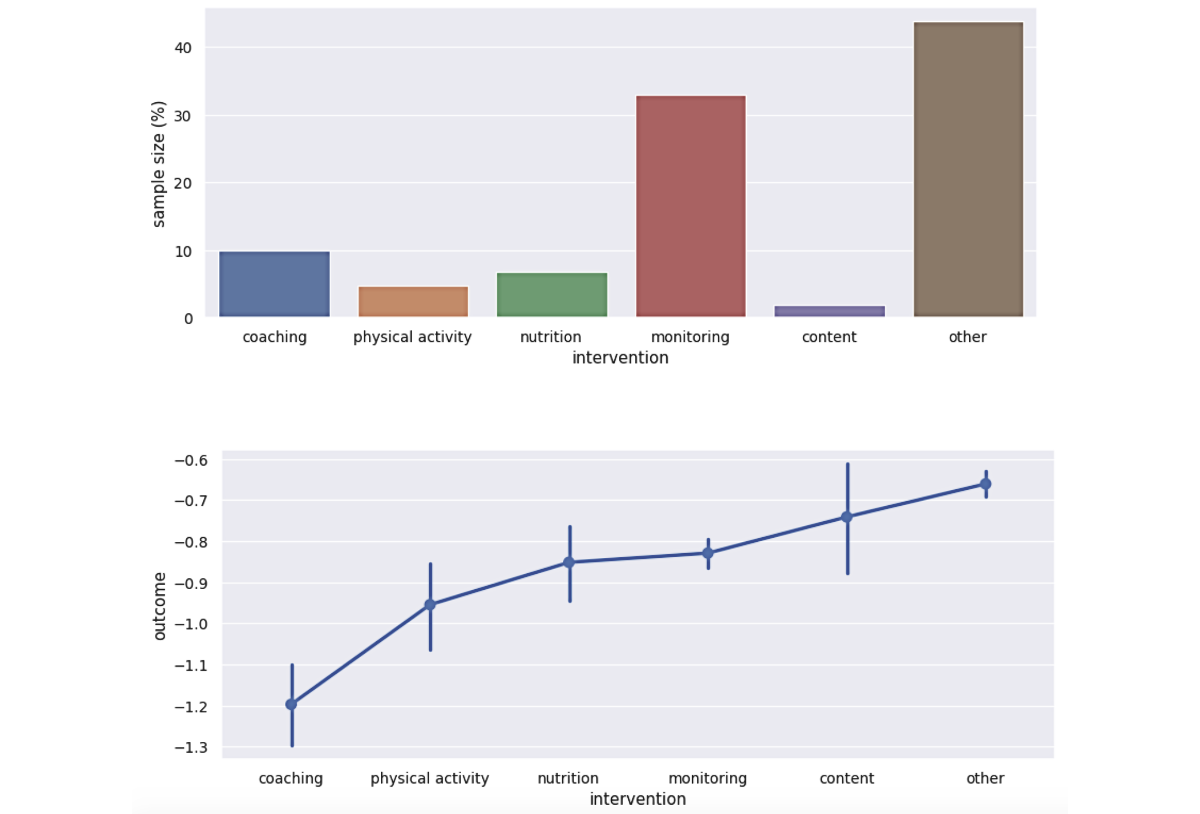
Top: distribution of members in the treatment cohort within the five different intervention categories or other category as a fraction of total data set sample size. Bottom: outcome (difference in estimated A_1c_ level) for the different interventions.

Figure 6 displays the total number of members within the treatment cohort of each of the five action categories and outcome *Y* related to the action category. Members with insufficient engagement in all action categories are assigned to the “other” category or control cohort.

As shown in Figure 6, the action categories had varying sample sizes. This is a result of engagement rates affected by member preferences or desired support to manage their condition. Members who engaged with coaching and physical activity had better outcomes (ie, more negative change in estimated A_1c_).

### Evaluation of Heterogenous Treatment Effect Model for Five Action Categories

**Figure 7:**
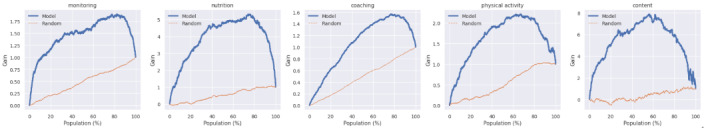
Cumulative gain of outcome for the five different interventions by model predictions (solid blue) and random sorting (dashed orange).

The model performances were evaluated using a common validation data set across interventions. The cumulative gain of the outcome when members are sorted by model predictions compared to random sorting for the five action category outcomes independently is shown in Figure 7. The area under the gain curve was larger when members were sorted by predicted treatment effect compared to random ranking, which confirmed that the model can infer causal effects of all the action categories in the data.

### Action/Intervention Recommendation Based on Heterogeneous Treatment Effect

**Figure 8:**
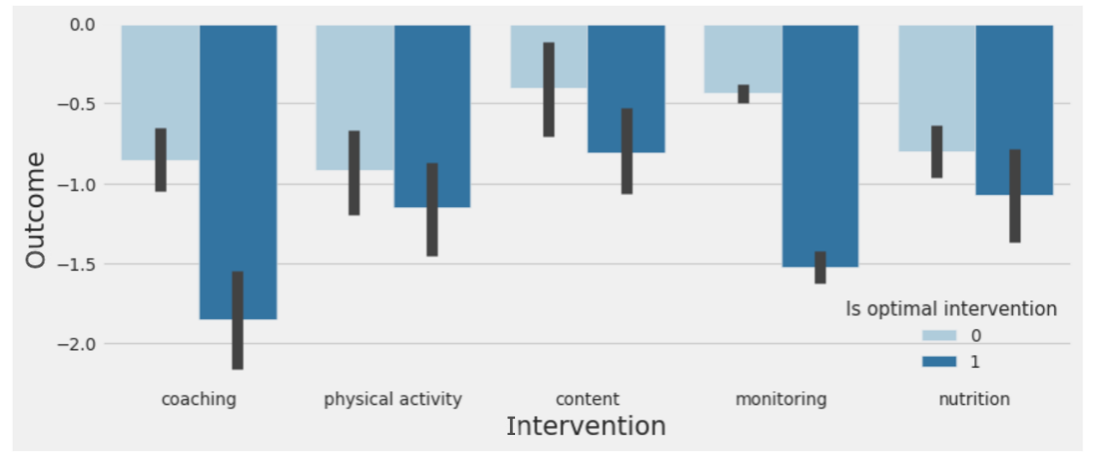
Change in estimated A_1c_ of members in different intervention treatment cohorts if the member received the model predicted optimal intervention (dark blue) or not (light blue).

For each member, the intervention with the most negative treatment effect is the action that the model predicted would result in a larger reduction in estimated A_1c_ (ie, optimal intervention). The average change in estimated A_1c_ for members who were part of the treatment cohort in at least one action category in the validation set is shown in dark blue if the received intervention was the same as the predicted intervention and shown in light blue if the received intervention was not the same as the predicted intervention (see Figure 8). Within all five action categories, the outcome is more negative when the prediction matches the true intervention, which indicated that the model was successful in identifying optimal outcomes. Members who participated in interventions that matched their optimal predictions had an estimated A_1c_ reduction of 1.4%, while those that did not participate in their predicted optimal intervention had a reduction of only 0.57%. This 0.8% estimated A_1c_ reduction difference can be attributed to intervention personalization. Members who had a predicted optimal intervention of coaching and received coaching showed the highest change in estimated A_1c_ at 1.7%.

The distribution of predicted optimal intervention for each member with negative predicted treatment effects were coaching (28%), SMBG checks (26%), physical activity (18%), content (16%), and nutrition (12%). Interaction with coaches and SMBG checks were observed to be the optimal intervention for 28% and 25% of the sample size, respectively, and the most recommended interventions. A balanced distribution of recommendations for optimal clinical outcomes was observed and opens an opportunity to prioritize recommendations based on a heterogeneous causal effect model.

**Figure 9:**
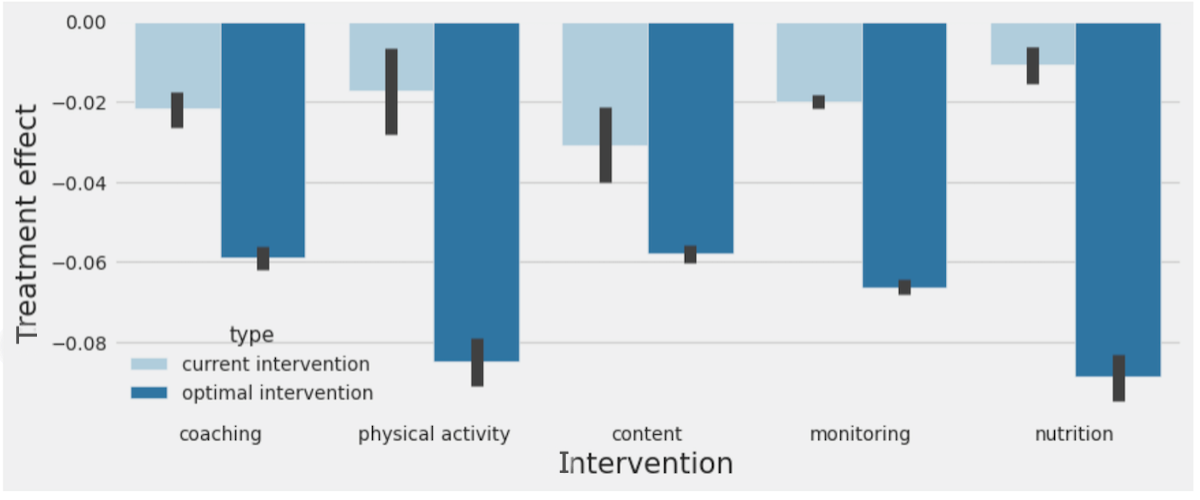
Computed treatment effect for members who received different interventions. The light blue bars denote the treatment effect for members who were in the treatment cohort of our data set. Dark blue bars denote treatment effect for members if they received the optimal intervention.

A comparison of average treatment effect with current interventions and with recommended optimal intervention predicted by the model is shown in Figure 9. Treatment effect was larger with the recommended optimal intervention for all action categories. On average, the recommended intervention predicts a treatment effect of 0.07% compared to 0.02%, with the current intervention producing a difference of 0.05% in estimated A_1c_ attributable to personalization.

## Discussion

This study highlights the feasibility of analyzing the engagement of members in an RDMP to develop a causal inference-based recommender system for predicting actions driving optimal clinical outcomes. Five action categories were identified upon member engagement level, and the causal inference model computed heterogenous treatment effect of each action per member. Model predictions were evaluated by comparing uplift gain when members were ranked by treatment effect to random sorting, and AUUC of the model predicted gain curves were larger for all actions, validating the method to infer treatment effect. Coaching and glucose monitoring were found to be the most frequently recommended actions for members to achieve optimal clinical outcomes. On average, members who engaged within their recommended actions had a 0.8% higher reduction in estimated A_1c_ than those who did not engage within recommended actions in their first 3 months of the program, with coaching showing the largest reduction in estimated A_1c_ at 1.7% when recommended and used by members.

Machine learning has been used to study precision medicine in diabetes care and complications, variables to predict the development of diabetes, and individual characteristics related to diabetes outcomes [20-22]. However, there is a lack of evidence around practical solutions for real-world implementation, specifically around diabetes self-management behaviors, which are the foundation of successfully living with the chronic condition [23]. It has been well-established that diabetes management programs are effective in helping participants obtain glycemic control, improve HbA_1c_ values, and increase self-efficacy with the support of diabetes coaches and structured SMBG [2,5,7-9]. Therefore, as real-world data has been made available around self-management behaviors through RDMPs, mobile apps, and other technologies, it is immensely valuable to use this data in machine learning techniques to provide personalized recommendations to enhance the future of diabetes care.

Our study observed better outcomes for members who engaged in their recommended actions over members who did not, across all action categories. On average, members who engaged within their recommended actions had a significant improvement estimated A_1c_ than those who did not engage within recommended actions. Therefore, we propose RDMPs develop recommended actions of engagement that are more likely to lead to better outcomes based on computed heterogeneous treatment effects with the most optimal action having the most negative treatment effect. By offering personalized recommendations, members can receive a more effective experience through both digital and human coaching allowing for not only a medical cost saving for the individual through improved health outcomes but also a more cost-effective approach for the RDMP by directing the member to the most valuable program features.

Type 2 diabetes management is complex and dependent upon many factors such as nutrition, physical activity, and medication adherence, which varies widely among this population as a whole; therefore, to generate successful personalized recommendations, all variables must be gathered to match an individual’s specific needs [24]. Our study presents one way that treatment effects can be computed from the causal model for recommending future interventions to drive clinical outcomes. Strong evidence around coaching, SMBG, education, nutrition, and physical activity has informed the development of diabetes management programs; however, the individual’s education gaps, lifestyle, available resources, and personal priorities get lost in the wide range of program features available. This could lead to overwhelm, burnout, and even distress from not knowing where one should place their focus. By offering our method to develop a recommender system for program feature engagement, we hope that guiding members to program features that are considered most effective in supporting clinical outcomes will lessen any burdens of disease management.

This study has several strengths, including the report of real-world data, as well as insight into the demographics and program engagement of members participating in an RDMP. Members were not provided incentives to participate in the program or study beyond the Livongo for Diabetes program being provided as a benefit through their employer or health plan package. The study also had some limitations, including the retrospective analysis study design. Members in the Livongo for Diabetes program received promotional engagement outreach in the form of mobile app nudges, emails, and text messages; therefore, observational data collected for the study contained a diverse set of engagement behaviors within the program features and did not provide a clean treatment and control cohort split. Improvement in estimated A_1c_ was calculated from participants’ SMBG values, which has been successfully correlated with laboratory HbA_1c_ values; however, it does have some limitations and is best used as a population-level tool.

Nonetheless, this study demonstrates how engagement thresholds that minimize modelling errors can be used to create control-treatment samples in observational data and compute treatment effects. The recommended action within the study is based solely on the likelihood of the member attaining a better outcome and member preferences, and propensity to engage in an action during prediction was not considered. Therefore, real-life implementation of the recommender system would have to include the likelihood of engagement and likelihood of outcome while personalizing the action recommendations. Although treatment effects were computed, it was assumed that the interventions were independent of each other and analyzed separately; however, using Bayesian inference of treatment effect would have accounted for dependencies between interventions and is recommend for a future study to further explore RDMP personalization.

Personalized action recommendations using heterogeneous treatment effects to compute the impact of member actions within an RDMP to significantly reduce estimated A_1c_ can be a valuable tool in driving member behaviors toward actions that are more likely to impact clinical outcomes. Future research is recommended to implement and evaluate this model prospectively within an RDMP.

## Abbreviations

AUUC: area under the uplift curve
BG: blood glucose
CDCES: certified diabetes care and education specialist
DR: doubly robust
HbA_1c_: hemoglobin A_1c_
MSE: mean squared error
RDMP: remote diabetes monitoring program
SMBG: self-monitoring of blood glucose
